# Bonwille’s Method of Rapid Breathing

**Published:** 1881-08

**Authors:** 


Dr. Addinell Hewson, of Philadelphia, is still getting excellent results from the use of Bonwille's method of rapid breathing in labor, and other cases, where anæsthesia is desired for short periods of time. He regards the process of rapid breathing necessary to producing insensibility to pain, as devoid of danger, or ill effects even; and his experience has extended over a period of several years, and has, probably, been much larger than that of any other practitioner in its use. The process by which analgesia, or anæsthesia, is effected is exceedingly simple, and can be practised in any case when the patient is conscious, and sufficiently intelligent to understand the purpose of the practitioner in wishing to bring it about. A napkin or towel is placed over the face of the patient, and rapid respiration directed—to be kept up until the object for which it is desired is accomplished. The immunity from pain is complete in many instances, and all seem to be relieved from acute suffering whilst the rapid breathing is continued.

